# The Dysregulation of Polyamine Metabolism in Colorectal Cancer Is Associated with Overexpression of c-Myc and C/EBP*β* rather than Enterotoxigenic* Bacteroides fragilis* Infection

**DOI:** 10.1155/2016/2353560

**Published:** 2016-06-28

**Authors:** Anastasiya V. Snezhkina, George S. Krasnov, Anastasiya V. Lipatova, Asiya F. Sadritdinova, Olga L. Kardymon, Maria S. Fedorova, Nataliya V. Melnikova, Oleg A. Stepanov, Andrew R. Zaretsky, Andrey D. Kaprin, Boris Y. Alekseev, Alexey A. Dmitriev, Anna V. Kudryavtseva

**Affiliations:** ^1^Engelhardt Institute of Molecular Biology, Russian Academy of Sciences, Moscow 119991, Russia; ^2^Orekhovich Institute of Biomedical Chemistry, Russian Academy of Medical Sciences, Moscow 119121, Russia; ^3^National Medical Research Center of Radiology, Ministry of Healthcare of the Russian Federation, Moscow 125284, Russia; ^4^Shemyakin-Ovchinnikov Institute of Bioorganic Chemistry, Russian Academy of Sciences, Moscow 117997, Russia

## Abstract

Colorectal cancer is one of the most common cancers in the world. It is well known that the chronic inflammation can promote the progression of colorectal cancer (CRC). Recently, a number of studies revealed a potential association between colorectal inflammation, cancer progression, and infection caused by enterotoxigenic* Bacteroides fragilis* (ETBF). Bacterial enterotoxin activates spermine oxidase (SMO), which produces spermidine and H_2_O_2_ as byproducts of polyamine catabolism, which, in turn, enhances inflammation and tissue injury. Using qPCR analysis, we estimated the expression of* SMOX* gene and ETBF colonization in CRC patients. We found no statistically significant associations between them. Then we selected genes involved in polyamine metabolism, metabolic reprogramming, and inflammation regulation and estimated their expression in CRC. We observed overexpression of* SMOX*,* ODC1*,* SRM*,* SMS*,* MTAP*,* c-Myc*,* C/EBPβ* (*CREBP*), and other genes. We found that two mediators of metabolic reprogramming, inflammation, and cell proliferation c-Myc and C/EBP*β* may serve as regulators of polyamine metabolism genes (*SMOX, AZIN1, MTAP, SRM, ODC1, AMD1*, and* AGMAT*) as they are overexpressed in tumors, have binding site according to ENCODE ChIP-Seq data, and demonstrate strong coexpression with their targets. Thus, increased polyamine metabolism in CRC could be driven by c-Myc and C/EBP*β* rather than ETBF infection.

## 1. Introduction

Colorectal cancer (CRC) is one of the most common cancers in the world. It has been estimated that in 2012 about 1.4 million people were diagnosed and more than 690 thousand died [1]. The lifetime risk of developing colorectal cancer is about 5% worldwide. Women have a higher risk for colon cancer that men [2]. The prognosis of colorectal cancer is closely related to the stage of disease at diagnosis [3]. CRC can have no symptoms in early stages, and mean 5-year survival rate for peoples detected at an early stage is about 90% compared to 10% for people diagnosed for cancer with distant metastases [4]. Certain factors increase a risk of developing the disease. These are age [5], polyps of the colon [6], history of cancer, heredity [7–9], smoking [10], diet and microbiota [11, 12], lack of physical activity [13], chronic inflammation (colitis and IBD) [14, 15], viruses [16, 17], and exogenous hormones [18]. The chronic inflammation caused by infection is one more risk factor for colorectal cancer [19, 20]. Some pathogenic strains of* Escherichia coli* (cyclomodulin-positive) are able to induce chronic inflammation and can be involved in carcinogenesis. Cyclomodulin-positive* E. coli* strains were more prevalent in both the mucosa and tumors of patients with colorectal cancer (26% patients) versus diverticulosis control (6% patients) [19]. In addition, the number of colonic polyps was elevated in multiple intestinal neoplasia (Min) mice inoculated with a colon cancer-associated* E. coli* strain (11G5) [19].

Recent studies have demonstrated that the enterotoxigenic* Bacteroides fragilis* (ETBF) bacterium is an important cause of chronic inflammation in human and animal colon. It has been presented that the* bft* gene, which encodes* Bacteroides fragilis* toxin (BFT), is associated with colorectal neoplasia and may be a risk factor for developing CRC [21]. It was observed that BFT produced by bacteria upregulates both spermine oxidase (*SMOX*) gene expression at mRNA and protein levels in cultures of human normal colonic epithelial cells [20].* SMOX* encodes SMO protein, which plays an important role in the regulation of polyamine metabolism. SMO catalyzes the oxidation of spermine to spermidine and produces hydrogen peroxide (H_2_O_2_) and aldehydes [22]. This results in apoptosis, DNA damage, and consequently the development of cancer. For example, cytotoxin produced by* Helicobacter pylori* strains causes an increase in spermine oxidase levels in human gastric epithelial cells. These pathogenic* H. pylori* strains contain cytotoxin-associated gene A (CagA) and represent a risk factor for gastric cancer. The strong association of* H. pylori* (Cag+) infection, SMO levels, apoptosis, and oxidative DNA damage has been observed [23, 24].

In recent years, we have seen a steady increase in the number of studies examining the role of intracellular polyamine metabolism in tumor development. Several important enzymes, spermidine/spermine N1-acetyltransferase (SSAT), N1-acetylpolyamine oxidase (APAO), and SMO, appear to play critical roles in many cancers. All such enzymes are highly inducible by multiple stress signals, including ones caused with bacterial pathogens, and have the potential to alter polyamine homeostasis. APAO and SMO enzymes produce reactive oxygen species (ROS), H_2_O_2_, and aldehydes, which are potentially harmful to cells. ROS are key signaling molecules, which play an important role in several pathways (e.g., NF-*κ*B, ERK1/2, p38, PI3K, and others) and can contribute to the induction of inflammation and cancer [25].

Thus, the abundant pathogenic microbiota alters the host tissue microenvironment leading to chronic inflammation, immune dysregulation, and elevated levels of ROS. All these may result in activation of oncogenes, downregulation of tumor suppressor genes, DNA damage, and cell and tissue injury, thereby contributing to tumor growth. In the colon, the alteration of polyamine catabolism caused by infection with consequent H_2_O_2_ generation and DNA damage may be a common cause of inflammation and promotion of carcinogenesis. Moreover, an increase in polyamine catabolism rates and the production of H_2_O_2_ has been involved in the response to chemotherapeutic agents or specific antitumor polyamine analogues in several tumors, including colorectal cancer [26–30]. However, the clinical, molecular, and prognostic associations of infection and the expression of polyamine metabolism gene in colorectal cancer remain unclear.

## 2. Material and Methods

### 2.1. Tissue Specimens

A total of 50 paired specimens of stages I–IV colorectal cancer (CRC) and adjacent morphologically normal tissues were taken from patients with primary carcinoma of the colon and rectum, which had not been exposed to radiation or chemotherapy, during surgical resection. Each sample was frozen and placed in liquid nitrogen immediately after surgery. The specimens were characterized according to the American Joint Committee on Cancer (AJCC) staging system [31]. The diagnosis was verified by histopathology and only samples containing 70–80% or more tumor cells were used in the study. The tissue samples were collected in accordance with the guidelines issued by the Ethics Committee, National Medical Research Radiological Center, the Ministry of Health of the Russian Federation. All patients gave written informed consent, which is available upon request. The study was carried out in accordance with the principles outlined in the Declaration of Helsinki (1964).

### 2.2. RNA and DNA Isolation and cDNA Synthesis

Total RNA was isolated using Micro-Dismembrator S (Sartorius, Germany) and RNeasy Mini Kit (Qiagen, Germany) in accordance with the manufacturer's instructions. For the detection of bacteria, DNA was extracted using the QIAamp DNA Mini Kit (Qiagen, Germany) and further treated with proteinase K in accordance with the manufacturer's protocol. Purified RNA and DNA were quantified using Qubit 2.0 fluorometer (Invitrogen, USA) and their quality was determined by Agilent Bioanalyzer 2100 (Agilent Technologies, USA). All RNA samples were treated with DNase I (Thermo Fisher Scientific, USA), and cDNA was synthesized using M-MLV Reverse Transcriptase (Thermo Fisher Scientific, USA) and random hexamers according to standard manufacturer's protocol.

### 2.3. qPCR

To detect and quantitate* Bacteroides fragilis* we used the primers targeting* Bft1* gene. These primers were taken from the work of Viljoen et al. [32]: forward 5′-GACGGTGTATGTGATTTGTCTGAGAGA-3′, reverse 5′-ATCCCTAAGATTTTATTATCCCAAGTA-3′. EvaGreen Dye (Biotium Inc., USA) was used as fluorescent DNA-binding dye for the detection and quantification of PCR products. Purified bacterial control DNA was obtained from Orekhovich Institute of Biomedical Chemistry, Russian Academy of Sciences (Moscow).

To evaluate gene expression, we used TaqMan Gene Expression Assays (Thermo Fisher Scientific, USA). This consists of a pair of specific unlabeled PCR primers and a TaqMan probe with a FAM dye label on the 5′ end and minor groove binder (MGB) nonfluorescent quencher (NFQ) on the 3′ end. All probes contained the dye FAM at 5′-end and RTQ1 at 3′-end. qRT-PCR was performed as described earlier using* RPN1* and* GUSB* reference genes [33–35].

All reactions were performed using AB 7500 Real-Time PCR System (Thermo Fisher Scientific, USA) with RQ (Relative Quantitation) software (Thermo Fisher Scientific, USA). PCR program was as follows: 10 min at 95°C and then 50 two-step cycles 15 s at 95°C and 60 s at 60°C. The total reaction volume was 20 *μ*L in triplicate. PCR products were analyzed in 2% agarose gels, and nucleotide sequences of the amplicons were verified by Sanger sequencing with ABI Prism 3100 Genetic Analyzer (Thermo Fisher Scientific, USA).

### 2.4. Analysis of qRT-PCR Data

For the detection and quantification of bacterial DNA, absolute quantification method was used. A standard curve was constructed using serial 10-fold dilutions of control bacterial DNA. The genome size of* Bacteroides fragilis* (5.3 Mb) and the mass of DNA per genome were used to calculate the concentration of bacterial DNA [36]. Spearman's rank correlation analysis was used to check the dependence between target gene expression levels and the concentration of bacterial DNA within the same samples.

mRNA qRT-PCR data were analyzed using the relative quantification method (ΔΔC_t_) taking into account the efficiency of the PCR amplification using ATG (Analysis of Transcription of Genes) tool as described in [34, 37]. The relative inner variability between mRNA levels of reference genes (*RPN1* and* GUSB*) was not higher than twofold in tumor and normal tissues; therefore, twofold and higher mRNA level changes for the target genes were considered as significant. We used nonparametric Mann-Whitney *U* test to validate the significance of gene expression alterations (*p* ≤ 0.01 was taken as the criterion of statistical significance). All statistical analyses were performed in the R environment. Pearson correlation coefficient was used to evaluate the coexpression of human genes (*p* ≤ 0.001 was taken as the criterion of statistical significance). We supplemented the coexpression analysis with ENCODE ChIP-Seq data using previously developed CrossHub tool [38].

## 3. Results

### 3.1. Expression of SMOX Gene and* Bacteroides fragilis* Quantification

**Figure 1:**
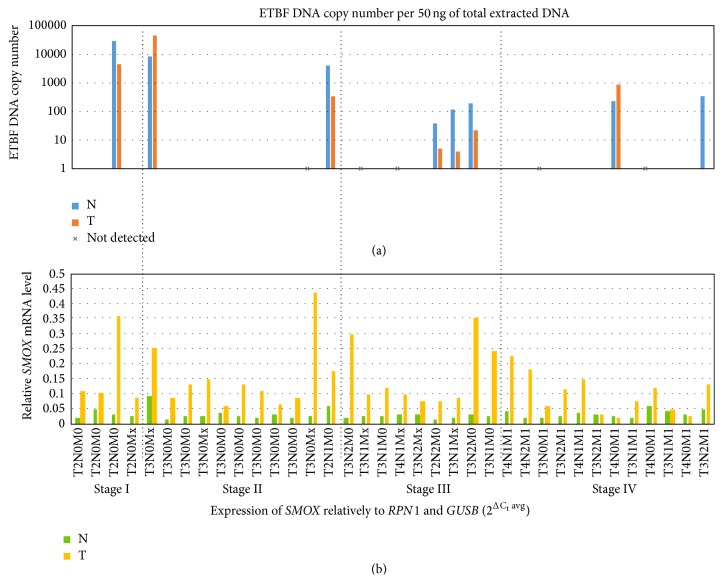
(a) Enterotoxigenic* B. fragilis* (ETBF) DNA copy number per 50 ng of total extracted DNA in paired samples of colorectal cancer (logarithmic scale). (b)* SMOX* expression level relatively to two reference genes:* RPN1* and* GUSB*. The samples with high rates of ETBF colonization tend to have higher expression of SMOX, especially in normal tissue (compared to the other norms). However, no statistically significant correlation between* SMOX* expression and ETBF colonization was observed.

We analyzed 36 paired samples of primary colorectal carcinomas and adjacent normal tissues to quantify ETBF. Serial dilution of genomic DNA from ETBF was used as standard. At least one copy of ETBF DNA per 50 ng of total extracted DNA was detected in 8 paired tumor and adjacent normal samples, including three samples, which showed a significantly increased amount of the ETBF DNA (>1000 copies/1 ng of extracted DNA). Most of the tested samples (23/36) demonstrated less than a copy of bacterial DNA per 50 ng of total extracted DNA. Five samples were found to be ETBF-negative (Figure 1).

### 3.2. Expression Quantification of Genes Involved in Polyamine Metabolism and Regulation of Inflammatory Response

The set of 36 colorectal tumors and adjacent normal tissues was extended with an additional set of 14 paired samples in order to increase the statistical significance of the results. Thus, we used a set of 50 colorectal cancers to evaluate the expression of target genes, which encode enzymes of polyamine metabolism and genes participating in immune response and inflammation signaling.

**Figure 2:**
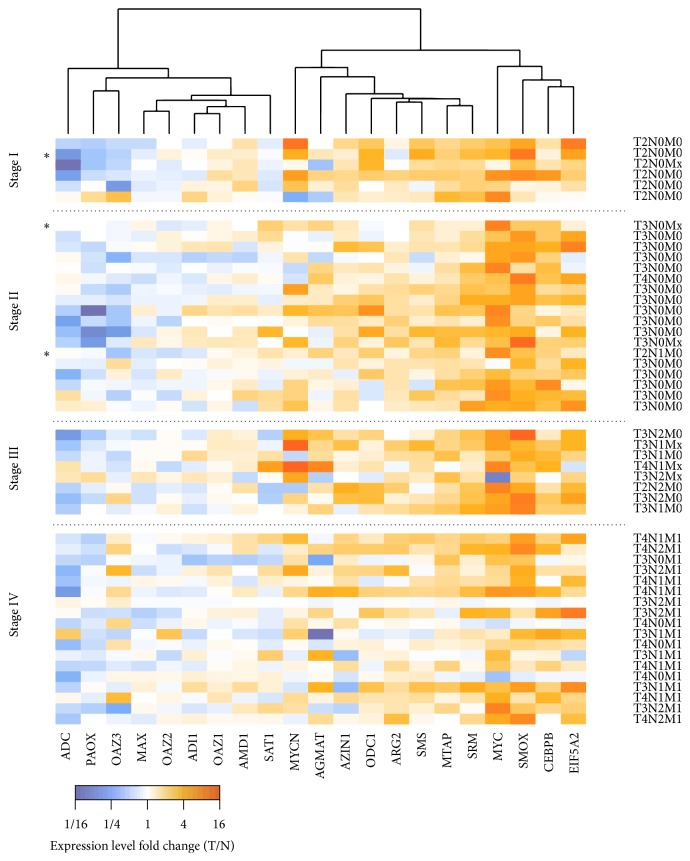
Results of the qPCR expression analysis of genes involved in polyamine metabolism and inflammation regulation in paired colorectal cancer samples. Cell color indicates expression level change in tumor compared to normal: increase (orange) and decrease (blue). Genes are rearranged according to the similarity of expression profiles. Samples with a high concentration of enterotoxigenic* B. fragilis* DNA (>1000 copies per 1 ng of total DNA) are marked with an asterisk.

**Figure 3:**
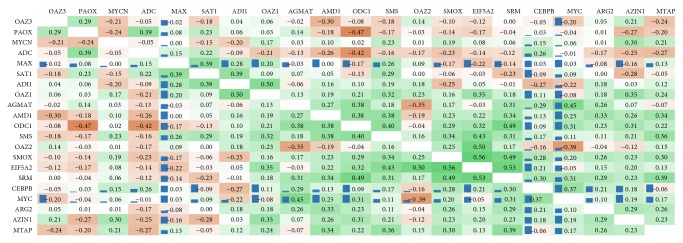
Results of coexpression analysis for genes participating in polyamine metabolism. Pearson correlation coefficients between the expression levels changes of genes participating in polyamine metabolism and inflammation across 50 colorectal cancer samples are presented. Cell color reflects these values (green: positive, brown: negative). Normalized ChIP-Seq score (according to ENCODE data) is indicated with blue bars.

Using RT-qPCR, we evaluated the relative expression level of 17 genes involved in polyamine metabolism and 4 genes mediating metabolic reprogramming, and the regulation of inflammatory response and cell proliferation:* c-Myc *(*MYC*),* n-Myc *(*MYCN*),* Max,* and* C/EBPβ* gene encoding CCAAT/enhancer binding protein (CEBPB). The results are given in Figure 2 (tumor/normal relative expression level) and Figure 3 (coexpression analysis coupled to ENCODE ChIP-Seq data).

Two genes,* c-Myc* and* SMOX*, demonstrated the highest upregulation in CRC. Both genes were overexpressed (at least 2 times) in 40 out of 50 samples (80%).* C/EBPβ*,* eIF5A2,* and* SRM* revealed upregulation in 50% samples. Several pairs of genes that demonstrated statistically significant coexpression were also found to have ChIP-Seq associations:* c-Myc-AGMAT, c-Myc-ODC1, c-Myc-SRM, c-Myc-AMD1, C/EBPβ-SRM, C/EBPβ-AGMAT, C/EBPβ-SMOX,* and* C/EBPβ-eIF5A2*. Moreover,* C/EBPβ*and* c-Myc* demonstrated strong coexpression (*r*_*s*_ = 0.37) and extremely high ChIP-Seq signal intensity according to ENCODE ChIP-Seq data (C/EBP*β* binding to c-Myc). This suggests C/EBP*β* to be a possible upstream regulator of* c-Myc*. In contrast to* c-Myc*,* n-Myc* showed statistically significant coexpression only with* AZIN1*.

## 4. Discussion

*Bacteroides fragilis* contains up to 1%-2% of the normal colonic microbial flora in humans [39, 40]. Pathogenic strains of* B. fragilis* that produce enterotoxin are associated with the development of inflammatory diarrheal disease in both children and adults [41, 42], IBD [43], and colitis [44], thereby contributing to chronic inflammation and onset of colon tumors. It was supposed that* B. fragilis* toxin (BFT), produced only by enterotoxigenic strains (ETBF), binds to a specific intestinal epithelial cell receptor and induces several signal transduction pathways [45, 46]. BFT cleaves E-cadherin [47, 48] and activates Wnt/*β*-catenin pathway signaling [49]. BFT was also shown to be involved in activation of tyrosine kinases, MAPKs, and NF-*κ*B signaling pathways and to be able to increase cellular proliferation mediated by the elevated expression of c-Myc oncogene [50–53]. Moreover, as mentioned above, BFT may be implicated in polyamine catabolism through upregulation of SMO protein levels and enzyme activity [20]. Recent studies suggested SMO enzyme as a potential source of an inflammation-associated ROS produced during polyamine catabolism [54]. Thus, the association between ETBF colonization rates of CRC patients and the expression of* SMOX* gene appears to be an important link between chronic inflammation caused by infection, tumor onset, and progression.

In this study, we quantitated ETBF strain colonization rates and evaluated the expression of SMOX gene at mRNA level in CRC patients using qPCR. We found that the majority of CRC patients were colonized with toxin-producing strains of* B. fragilis*, but only small amounts of bacterial DNA were identified. A significantly increased content of bacterial DNA was detected in three patients with I and II stages of CRC (in both tumor and normal tissues). In contrast, pathogenic cyclomodulin-positive* E. coli* strains, a possible cofactor of colorectal carcinogenesis, were predominantly found on mucosa of patients with stages III/IV [19]. Additional studies and extended sampling are needed to reveal possible associations between ETBF colonization and disease stage.

The expression of* SMOX* gene revealed no statistically significant correlation with the amount of bacterial DNA, but the samples with a high concentration of bacterial DNA (>1000 copies per 50 ng) demonstrated high SMOX expression levels in both normal tissue and tumor (relatively to* GUSB* and* RPN1* reference genes, Figure 1). These results do not contradict the hypothesis that ETBF strains can be proinflammatory and oncogenic bacteria, but additional studies are needed to understand the possible role of ETBF in colorectal carcinogenesis [21]. These results are consistent with a mechanism of* SMOX* induction independent of ETBF infection.

Next, we tested differential expression of 17 genes participating in polyamine metabolism and 4 genes involved in the mediation of metabolic preprogramming, cell proliferation (*с-Myc*,* n-Myc*, and* Max*) and inflammation (C/EBP*β*). c-Myc and n-Myc form dimers with Max, translocate to the nucleus, and then activate the transcription of many genes participating in cell cycle regulation, glycolysis, energy metabolism, hypoxic adaptation, DNA replication, and other processes [55–58]. C/EBP*β* is a transcription factor, which can form either homodimers or heterodimers with other CCAAT/enhancer binding proteins (alpha, delta, and gamma). C/EBP*β* is known to be a mediator of inflammation and immunity [59, 60]. The most important C/EBP*β* targets are interleukins IL-6, IL-4, IL-5, and TNF-*α* [61–64]. We found upregulation of* c-Myc*,* n-Myc*, and* C/EBPβ* in the analyzed samples. However, we did not find overexpression of* Max*. This is in agreement with a previous finding: overexpression of* c-Myc* and* n-Myc* but not* Max* is observed in many tumors [55, 58, 65, 66]. Our data showed increased expression of* SMOX* gene at all stages of colorectal cancer, this tendency being more pronounced at the early stages (Figure 2). Spearman correlation coefficient between disease stage and tumor/normal fold change of* SMOX* mRNA level is *r*_*s*_ = −0.19:* SMOX* expression tends to be lower with disease stage. However, this tendency is not statistically significant (*p* > 0.05). Different tumors demonstrate dramatically distinct* SMOX* expression profiles. SMO was found to be elevated in both prostate adenocarcinoma and prostatic intraepithelial neoplasia [67] whereas breast cancer showed* SMOX* underexpression at both mRNA and protein levels [68]. Other genes that showed statistically significant correlation with disease stage were* MTAP* (*r*_*s*_ = −0.30, *p* = 0.03), and* SRM* (*r*_*s*_ = −0.27, *p* = 0.05), which tended to decrease the expression level with tumor stage, and* OAZ3* (*r*_*s*_ = 0.42, *p* = 0.003), which increased its expression with disease progression.

**Figure 4:**
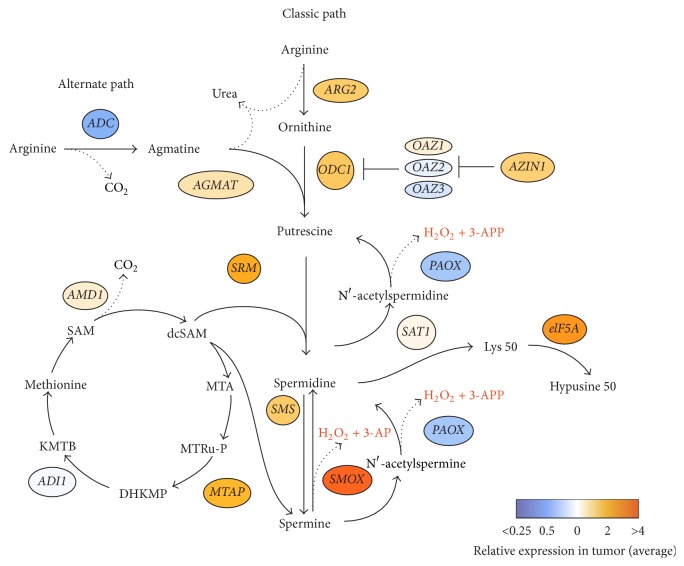
Classic path of polyamine metabolism consists of the following: (1) arginine is converted to ornithine through the action of ARG (arginase) in the urea cycle; (2) putrescine is formed from the reaction of ornithine decarboxylation catalyzed by ODC1 (ornithine decarboxylase-1). OAZ can bind to ODC1 to form OAZ-ODC1 complex and subsequently reduce polyamine synthesis. AZIN1 (antizyme inhibitor-1) brakes the ODC1-OAZ complex and liberates ODC1; (3) AMD1 (S-adenosylmethionine decarboxylase) decarboxylates S-adenosylmethionine (SAM) to decarboxylated SAM (dcSAM); (4) dcSAM provides aminopropyl groups to putrescine to produce spermidine by spermidine synthase (SRM) and spermine by spermine synthase (SMS). MTA (methylthioadenosine) is generated as a byproduct. Spermine can be recycled back to spermidine directly by spermine oxidase (SMOX). Spermine and spermidine can be recycled to spermidine and putrescine by spermidine/spermine-N1-acetyltransferase (SAT1) followed by oxidation by polyamine oxidase (PAOX) [101]. MTA can be processed to the methionine: MTA phosphorylase (MTAP) catalyzes the cleavage of MTA yielding 5-methylthioribose-1-phosphate (MTRu-P), which is further metabolized to DHKMP (1,2-dihydro-3-keto-5-methylthiopentene). ADI (acireductone dioxygenase) catalyzes DHKMP to 2-oxo-4-methylthiobutyrate (KMTB) and transamination of KMTB results in formation of methionine [102–104].

Polyamines are organic cations, which are essential for cell proliferation and growth, and their levels are frequently altered in many human tumors [69, 70]. Polyamines have also been shown to play an important role in inflammation-induced carcinogenesis [71]. Intracellular polyamine metabolism occurs via two pathways: classical and alternate (Figure 4). Polyamines (putrescine, spermidine, and spermine) are synthesized by mammalian cells, while agmatine is produced only by plants and bacteria, including intestinal microbial flora [72–74]. L-arginine carboxylase (ADC), found only in nonmammals, catalyzes a reaction of decarboxylation of L-arginine to agmatine [72, 75]. The latter is further hydrolyzed to putrescine and urea by agmatinase, which is encoded by* AGMAT* gene. Recent reports also supposed that different pathogens, such as viruses and bacteria, could upregulate agmatinase at mRNA and protein levels, thereby inducing polyamine synthesis [76, 77].

In the present study, we revealed that the expression of* ADC* gene decreased on the average by half in CRC samples compared to adjacent normal tissues. These findings indicate that, in case of colorectal cancer, the enhanced polyamine synthesis is not associated with alternate path and the intestinal microbiota does not significantly contribute to these processes.

The three major enzymes that are involved in polyamine metabolism (SSAT, APAO, and SMO) are encoded by* SAT1, PAOX*, and* SMOX* genes, respectively. One of the products of both SMO and APAO-mediated reactions are H_2_O_2_ and highly toxic aldehydes, which may also cause oxidative damage. SSAT cellular toxicity is thought to be caused by produced acetylated polyamines, which are utilized by APAO. APAO is constitutively expressed, while SSAT is an inducible enzyme [78]. SSAT expression is induced by different stimuli, such as toxins, hormones, cytokines, nonsteroidal anti-inflammatory agents, natural products, and pathogens. It is regulated via several pathways including TNF-*α* and NF-*κ*B [79–82]. SSAT expression can be also mediated by Nrf2 in response to the addition of H_2_O_2_ in human hepatoma HUH7 cells [83]. We found that* SAT1* gene was differentially expressed in many CRC samples (either up- or downregulated), while a decreased expression of* PAOX* gene was observed. This indicates that the SSAT/APAO pathway is not hyperactive and does not seem to be a cause of oxidative damage in colorectal cancer. Inflammatory response agents and stress pathways are not the result of the induction of* SSAT* expression at mRNA level. Thus, it is possible that the increased* SMOX* gene expression and enzyme activity make the greatest contribution to the oxidative stress damage caused by polyamine catabolism in colorectal cancer [20].

It is known that specific protein products of oncogenes and tumor suppressor genes can regulate polyamine metabolism [84–86]. The results of coexpression analysis coupled to ENCODE ChIP-Seq data strongly suggest c-Myc and C/EBP*β* as regulators of the expression of key enzymes of polyamine metabolism that are upregulated in colorectal cancer:* SMOX, AZIN1, MTAP, SRM, AMD1, ODC1*, and* AGMAT*. It should be mentioned that genes encoding polyamine metabolic enzymes are regulated transcriptionally/post-transcriptionally by changes in the levels of intracellular polyamines. Additional studies (*c-Myc* and* C/EBPβ* knockdown assays) are needed to prove our finding. Not surprisingly, three of these genes,* ODC1, AMD1*, and* SRM*, are already known c-Myc targets [87–89]. Besides these genes, c-Myc is also known to regulate* eIF5A2*. However, this gene did not demonstrate coexpression with* c-Myc* but did show ChIP-Seq association (c-Myc binding site). It is known that c-Myc induces the expression of ornithine decarboxylase (ODC), which catalyzes the first rate-limiting step in polyamine synthesis [89]. It has been also shown that bacterial infections can stimulate polyamine synthesis through ODC [90], whereas suppression of ODC leads to the depletion of cellular polyamine levels in human colorectal cancer cells [82, 91]. ODC inhibition with difluoromethylornithine (DFMO) is a possible anticancer therapy. It was reported that the treatment of normal intestinal epithelial cells of rats (IEC-6) with the DFMO led to the depletion of polyamines and subsequently inhibited cell growth and induced cell cycle arrest. A combination of drugs including DFMO could reduce recurrent adenomatous polyps in patients with history of resected sporadic colorectal adenomas [92].

n-Myc (*MYCN*) is oncogenic transcription factor, which can directly upregulate ODC expression in neuroblastomas [93]. Moreover, it has been shown that the reduction of n-Myc protein levels through inhibition of glycolysis may decrease ODC expression and potentiate polyamine levels in human neuroblastoma cell lines [84]. We showed that expression of* ODC1* gene was increased in a number of CRC samples, as well as mRNA level of* c-Myc* and* n-Myc* genes. However,* n-Myc* showed no expression correlations with* ODC1* and no ENCODE ChIP-Seq data are available for n-Myc.

It is worthy of note that the expression of* OAZ1, OAZ2, *and* OAZ3* genes, which encode major regulators of ornithine decarboxylation to putrescine through inhibition of ODC catalytic activity, and the expression of* AZIN1* gene, which is involved in inhibition of antizyme (OAZ) family, did not change in the majority of CRC samples compared with normal samples. This indicates that the production of putrescine, catalyzed by ODC during polyamine catabolism, is not suppressed in colorectal cancer.

We have observed a significant increase in* eIF5A2* mRNA levels in colorectal tumors. Cytosolic protein encoded by* eIF5A2* gene undergoes posttranslational modification of Lys 50 to hypusine [94]. Spermidine as a substrate is involved in the first step of this process. The eIF5A2 protein is essential for eukaryotic cell proliferation, but the molecular function of eIF5A remains incompletely clear. It was shown that c-Myc can possibly transactivate the* eIF5A2* gene [95, 96]. eIF5A2, in turn, was demonstrated to regulate MTA1 (metastasis-associated 1) via c-Myc in gastric cancer and colorectal carcinoma [97, 98]. Our data suggests that eIF5A2 can play an important oncogenic role in CRC and enhance the involvement of polyamines in this pathological process.

We elevated the expression of* C/EBPβ* gene, an important transcription factor, which controls the expression of genes involved in inflammatory response [60, 99, 100]. We observed a positive correlation between* C/EBPβ* gene expression and many key genes such as* ADC, SMOX, AGMAT,* and* SRM* involved in polyamine metabolism.

## 5. Conclusions

In summary, our results show that dysregulation of polyamine metabolism in all stages of CRC can be associated with chronic inflammation mediators rather than with the infection caused by ETBF. This is the first report that presents changed levels of expression of the key components of polyamine metabolism. We characterized some important aspects of the expression of* SMOX* and* PAOX* genes, which are responsible for cellular ROS generation. Two transcription factors, oncogenic с-Myc (responsible for metabolic reprogramming and cell proliferation) and C/EBP*β* (mediator of inflammation and immune response), were found to be the most likely regulators of several key enzymes of polyamine metabolic pathway.
